# Atrial cardiopathy and stroke

**DOI:** 10.1055/s-0042-1758385

**Published:** 2022-11-09

**Authors:** Mitchell S. V. Elkind

**Affiliations:** 1 Columbia University, Vagelos College of Physicians and Surgeons, Department of Neurology, New York, United States.; 2 Columbia University, Mailman School of Public Health, Department of Epidemiology, New York, United States.

Paroxysmal atrial fibrillation (AF) is an important cause of cryptogenic stroke. AF is important to identify because oral anticoagulants substantially reduce risk of recurrence in patients with AF. Because AF may be asymptomatic, intermittent, and infrequent, however, many stroke patients have no evidence of AF during a brief hospital stay.

Because of these challenges, long-term cardiac monitoring is often employed to detect occult AF after stroke. Approximately 12% of patients with cryptogenic stroke–also called embolic stroke of undetermined source (ESUS) when a thorough evaluation has excluded alternate causes–may be found to have AF at one year. 1 Rates of AF are similar in those with small vessel and large vessel strokes. 2 Cardiac monitors may not detect AF for months or years after stroke, however. Thus, reliable predictors at time of stroke of the likelihood of detecting AF would be extremely valuable.

Earlier studies showed that detection of AF after stroke can be predicted by patient history, electrocardiographic indices like prolonged PR interval, 3 or biomarkers, like Brain Natriuretic Peptide (BNP). 4 As of yet, however, the ideal biomarkers have not been established. In this issue of *Arquivos de Neuro-Psiquiatria* , Çinar and colleagues provide evidence that electrocardiographic parameters, and particularly P wave measurements reflecting atrial conduction, may reliably predict which patients with stroke will develop AF. 5 The investigators explored P wave peak time (PWPT), P wave duration (PWD), and P wave amplitude in the first anterior chest lead among 231 consecutive acute ischemic stroke cases. Over a median follow-up time of 16 months, AF was detected in 43 cases, almost 20% of patients, similar to the studies noted above. All studied P wave parameters were found to be associated with AF detection in univariable analyses. In multivariable analysis, dementia, left atrium volume index, PWD, PWPT, and advanced interatrial block morphology all independently associated with detection of AF. PWD was the strongest predictor among P wave parameters. Further research is needed to confirm the role of P wave duration in AF prediction after stroke in diverse cohorts; to determine the optimal combination of clinical, electrocardiographic and other parameters to predict AF; and to assess clinical implications of these measures.

Biomarkers that predict AF may also be considered indicators of a condition termed atrial cardiopathy. Recent evidence, for instance, challenges the simple notion that stasis in a fibrillating atrium—the so-called “bag of worms”—determines stroke risk. Studies among large numbers of cardiac patients with implanted monitoring devices, for example, provide evidence of temporal disassociation: only about a quarter of patients were in AF in the 30 days prior to stroke. 6 Fibrosis and other acquired abnormalities of the left atrium, even in absence of AF, may provide a milieu for thrombus formation by promoting stasis, endothelial dysfunction, and hypercoagulability.

At present, there is no consensus on how to define, diagnose, or treat atrial cardiopathy. Several atrial biomarkers, however, may help assess stroke risk. Moderate to severe left atrial enlargement (LAE) independently predicts recurrent stroke. 7 P-wave terminal force in lead V1 (PTFV1), assessed on a standard ECG, and N-Terminal pro-BNP, are other markers of atrial dysfunction associated with stroke risk, even in patients without known AF. Among 3,723 participants free of both stroke and AF at baseline, PTFV1, NT-proBNP, and incident AF were each independently associated with incident stroke. 8 These findings suggest that electrocardiographic and serum biomarkers may be early correlates of atrial cardiopathy, occurring even prior to structural changes in the atrium.

Emerging evidence also suggests that patients with atrial cardiopathy without evidence of AF may be optimally treated with anticoagulants to prevent recurrent stroke, as is standard for AF patients. In post-hoc analysis of the Warfarin Aspirin Recurrent Stroke Study, for patients with NT-proBNP >750 pg/ml, treatment with warfarin was associated with reduced stroke or death risk at 2 years when compared to aspirin. 9 Similarly, in secondary analysis of NAVIGATE-ESUS (New Approach Rivaroxaban Inhibition of Factor Xa in a Global Trial versus ASA to Prevent Embolism in Embolic Stroke of Undetermined Source), anticoagulation was of greater benefit than aspirin among patients with LAE. 10 These hypothesis-generating results provide a rationale for conducting a trial testing anticoagulation among patients with unexplained stroke and atrial cardiopathy.

The Atrial Cardiopathy and Antithrombotic Drugs in Prevention after Cryptogenic Stroke Trial (ARCADIA; clinicaltrials.gov NCT03192215), supported by the US National Institute of Neurological Disorders and Stroke (NINDS), with ancillary support from the BMS-Pfizer Alliance for Eliquis® and Roche, is just such a trial. 11 ARCADIA will test the hypothesis that the anticoagulant apixaban is superior to aspirin for prevention of recurrent stroke in patients with cryptogenic stroke and atrial cardiopathy, but without known AF. All patients must have standard diagnostic testing to exclude causes of stroke, including AF. Eligible participants must then demonstrate one of three biomarkers of atrial cardiopathy: (1) PTFV _1_ > 5,000 μV*ms on 12-lead ECG; (2) serum NT-proBNP >250 pg/mL; or (3) left atrial diameter index ≥3 cm/m ^2^ on echocardiogram (severe LAE). The primary efficacy endpoint is recurrent stroke of any type. Results are expected in late 2024.

Atrial cardiopathy may constitute one of the mechanisms of unexplained stroke, but the best biomarkers to establish its presence remain uncertain. The data from Çinar and colleagues, ARCADIA, and other studies, provide evidence that future definitions are likely to include P wave parameters. If so, neurologists in the future may use these biomarkers to determine appropriate therapy and reduce recurrent stroke in their patients.
